# Phenolic Release during In Vitro Digestion of Cold and Hot Extruded Noodles Supplemented with Starch and Phenolic Extracts

**DOI:** 10.3390/nu14183864

**Published:** 2022-09-18

**Authors:** Ruibin Wang, Ming Li, Margaret Anne Brennan, Don Kulasiri, Boli Guo, Charles Stephen Brennan

**Affiliations:** 1 Key Laboratory of Agro-Products Processing, Institute of Food Science and Technology, Chinese Academy of Agriculture Sciences, Ministry of Agriculture and Rural Affairs, Beijing 100193, China; 2 Department of Wine, Food and Molecular Biosciences, Faculty of Agriculture & Life Sciences, Lincoln University, Lincoln 7647, New Zealand; 3 Riddet Institute, Massey University, Palmerston North 4474, New Zealand; 4 School of Science, Royal Melbourne Institute of Technology University, Melbourne, VIC 3000, Australia

**Keywords:** extrusion, starch, complexes, in vitro digestion, phenolic release

## Abstract

Dietary phenolic compounds must be released from the food matrix in the gastrointestinal tract to play a bioactive role, the release of which is interfered with by food structure. The release of phenolics (unbound and bound) of cold and hot extruded noodles enriched with phenolics (2.0%) during simulated in vitro gastrointestinal digestion was investigated. Bound phenolic content and X-ray diffraction (XRD) analysis were utilized to characterize the intensity and manner of starch-phenolic complexation during the preparation of extruded noodles. Hot extrusion induced the formation of more complexes, especially the V-type inclusion complexes, with a higher proportion of bound phenolics than cold extrusion, contributing to a more controlled release of phenolics along with slower starch digestion. For instance, during simulated small intestinal digestion, less unbound phenolics (59.4%) were released from hot extruded phenolic-enhanced noodles than from the corresponding cold extruded noodles (68.2%). This is similar to the release behavior of bound phenolics, that cold extruded noodles released more bound phenolics (56.5%) than hot extruded noodles (41.9%). For noodles extruded with rutin, the release of unbound rutin from hot extruded noodles and cold extruded noodles was 63.6% and 79.0%, respectively, in the small intestine phase, and bound rutin was released at a much lower amount from the hot extruded noodles (55.8%) than from the cold extruded noodles (89.7%). Hot extrusion may allow more potential bioaccessible phenolics (such as rutin), further improving the development of starchy foods enriched with controlled phenolics.

## 1. Introduction

Dietary phenolic compounds have attracted increasing attention in modulating the blood glucose, cholesterol, and radical scavenging activity, against certain chronic diseases such as type-II diabetes, cardiovascular disease, obesity, and cancer [1,2,3]. However, in order for phenolic compounds to play a bioactive role in the human body, they must be incorporated into foods and released from the food matrix in the gastrointestinal tract [4].

As for phenolic-enhanced starchy foods, starch digestion is accompanied by the release of phenolic compounds. A slow digestion rate and a low digestibility of the starch matrix were related to a controlled release of phenolics [5,6]. Based on the in vitro starch digestion curve, Zhu, et al. [7] reported that epigallocatechin-3-O-gallate (EGCG) embedded on the surface of starch was released rapidly within the first 1 h of gastrointestinal digestion. EGCG embedded in the cross-section of starch granule was released slowly within the next 2 h, and the EGCG complexed with amylose and amylopectin was finally released and reached equilibrium after 3 h. Therefore, it is important to consider the different forms of phenolic compounds in a starchy matrix to understand their degradation properties. Research has illustrated that the starch-gallic acid complexes with a high degree of complexation (13.21 mg of gallic acid per gram of starch) released the highest cumulative content of gallic acid after 120 min of digestion compared with samples with a lower degree of complexation (4.54 mg and 11.24 mg of gallic acid per gram of starch) [5]. Additionally, the manner of starch-phenolic complexation interfered with the releasing behavior of phenolics. The starch-phenolic inclusion complexes, phenolics inclusive in the cavity of a helix of amylose (V-type inclusion complexes) [8], have been shown to have a slower digestion rate and lower digestibility than the non-inclusion complexes [8,9], possibly indicating a slower phenolic release from the inclusion complexes.

Different from a model system with mixed starch and phenolics in a gel [5], special foods with a structure and shape may have a varying release of phenolics [10]. With respect to noodles, our previous study has illustrated that the digestion of cold extruded noodles prepared with starch and phenolics (mainly physically wrapped in the starch matrix) increased the level of total phenolics released when comparing the estimates of release at the end of the gastric phase and the small intestine [11]. Compared to cold extrusion, hot extrusion can cause a higher degree of disrupted multi-level structure (such as crystalline structure and chains) [12,13] and a higher degree of gelatinization [14], thereby possibly inducing more phenolics to complex with gelatinized or degraded starch. The presence of unbound phenolics and starch-phenolic complexes may inhibit the digestion of extruded noodles by decreasing the activity of digestive enzymes or resisting the hydrolysis of enzymes, respectively [15,16,17,18,19,20], thus indicating a slower release of phenolics. However, an opposite consideration is that the gelatinized or disrupted starch induced by extrusion is likely to experience an accelerated digestion owing to the promoted susceptibility to enzyme hydrolysis [14], along with a faster release of phenolics. Therefore, it is worth investigating the release of phenolics in different forms during the digestion of extruded noodles. An assumption is that hot extruded noodles may experience a more controlled phenolic release than cold extruded noodles owing to an improved molecular complexation between starch and phenolics.

In this study, noodles supplemented with starch and phenolic extract (2%) with different forms of phenolics were prepared using cold and hot extrusion. Extruded noodles were in vitro digested, and the release of total phenolics, including predominant profiles, from the extruded noodles was monitored during digestion. A possible mechanism of phenolic release was introduced to assist the understanding of how the bound phenolics can be controlled during release, further improving their bioaccessibility.

## 2. Materials and Methods

### 2.1. Materials

Dehulled buckwheat flour was purchased locally (Ceres Organics, Auckland, New Zealand). Buckwheat hull was purchased from Farmers Mill Ltd. (Timaru, New Zealand). Pepsin (P6887, 3200–4500 U/mg), pancreatin (P7545 visceral, 8 U/mg), amyloglucosidase (AMG) (A7095, ≥260 U/mL) were from Sigma, New York, NY, USA. Folin-Ciocalteu reagent (2 N) was obtained from Sigma, St Louis, MO, USA. All chemicals used were of analytical grades.

### 2.2. Chemical Extraction

Starch was extracted from the buckwheat flour using the method described by Cho, et al. [21]. Since different components in buckwheat flour in water suspension tend to layer after centrifugation, the slurry of buckwheat flour was mixed with distilled water (1:5, *w*/*v*), and the starch was sedimented within 2 h. The excess water was dumped, followed by centrifugation at 3500× *g* for 10 min in a centrifuge (MULTIFUGE X3R, Thermo, Auckland, New Zealand). The brown layer on top of the pellet was scraped off. This process was repeated 5 times. The remaining pellet was lyophilized (ALPHA 1-2 LD plus, CHRIST, Germany) and ground using a grinder with a 500 µm sieve. The AACC Methods of 46-10 [22], 30-10 [23], and 08-01 [24] were used to measure the content of crude protein, crude fat, and ash of buckwheat flour, hull, and starch extract, respectively. The starch, dietary fiber, and resistant starch contents were measured using the total starch, dietary fiber, and resistant starch analysis kits from Megazyme International (Bray, Ireland).

Phenolic compounds were extracted from the buckwheat hull (with a higher content of phenolics than bran and flour [25]). Compared to conventional chemical extraction, ultrasound-assisted extraction can accelerate the extraction and improve the extraction efficiency [26,27]. The experimental conditions were selected based on the operational limits of the ultrasonic bath and the method described by Liu, et al. [28] with minor modification. Briefly, the buckwheat hull suspended in 70% ethanol (1:5, *w*/*v*) was subjected to ultrasound-assisted extraction (ultrasonic frequency of 59 KHz and power of 500 W) for 30 min and then centrifuged at 2500× *g* for 10 min. The supernatant was collected and evaporated to remove ethanol. Samples were freeze-dried and stored at −20 °C.

### 2.3. Preparation of Extruded Noodles

The phenolic extract (2%, based on starch weight) was pre-dissolved in distilled water, and then the buckwheat starch was blended with the solution to adjust the moisture content to 35%, followed by equilibration at 4 °C overnight. The blend was extruded into starch-phenolic noodles using a twin-screw extruder (DSE-25, Brabender OHG, Duisburg, Germany) with the ratio of screw length to diameter (L/D) of 20:1 and the diameter of the die nozzle was 3 mm. Cold (CE) and hot extrusion (HE) were carried out under the temperature of 40-40-40-40-40 °C (I–V zones) and the temperature of 40-65-90-90-90 °C (I–V zones), respectively. The screw speed was 100 rpm, and the feed rate was 20 g/min. Control samples were prepared using starch only. The buckwheat starch was blended with distilled water (the moisture content was 35%, dry-based), followed by equilibration at 4 °C overnight. The extrusion conditions of control samples were the same as those of phenolic-enriched noodles. The noodles were stored at −20 °C before analysis, considering the possible retrogradation of starch at room temperature or 4 °C [29] on cold or hot extruded starchy noodles.

### 2.4. X-ray Diffraction (XRD) Analysis

The crystalline structure of non-extruded starch and extruded noodle powders were monitored using an X-ray diffractometer (Ultima IV, Rigaku, Tokyo, Japan) using Cu-Kα radiation at 40 kV and 40 mA. The diffractograms were obtained over a scattering range (2θ) range of 5–40° at a scanning speed of 20° min^−1^ with a scanning step of 0.01° [30]. All measurements were undertaken in triplicate. Relative crystallinity (*RC*) and that of V-type crystallites (*RC*_v_) were calculated using the OriginPro 9.0.0 Software (OriginLab Corporation, Northampton, MA, USA) according to Equations (1) and (2). The proportion of V-type crystallinity (*P*_v_) was calculated according to Equation (3):

(1) $$\mathrm{RC}\;(\%)=\frac{A_{c}}{A_{c}+A_{a}}\times 100$$

(2) $${\mathrm{RC}}_{v}\;(\%)=\frac{A_{\mathrm{vc}}}{A_{c}+A_{a}}\times 100$$

(3) $$p_{v}\;(\%)=\frac{A_{\mathrm{vc}}}{A_{c}}\times 100$$

where A_c_, A_a_, and A_vc_ are the integrated areas of all the crystalline peaks, amorphous region, and V-type crystalline peaks on the XRD pattern.

### 2.5. Simulated In Vitro Digestion Analysis

Extruded noodles were in vitro digested as described previously [31]. Extruded noodles with 100 mg of starch (dry weight) were soaked in distilled water (6 mL) for 3 h in a water bath at 30 °C. The soaking for 3 h prior to cooking can shorten the optimal cooking time of noodles to 4 min with a relatively smooth surface. Afterward, the samples were boiled at 100 °C for 4 min and cooled to 37 °C in a water bath. The digestion process was activated when pepsin in 0.02 M hydrochloric acid (1 mg/mL, 5 mL) was added. The blends were incubated at 37 °C for 30 min with continuous stirring at 500 rpm. Then, sodium hydroxide (0.02 M, 5 mL), distilled water (2 mL), and mixed enzyme solution (2 mg pancreatin (16 U/mg), 7.9 mL of 0.2 M sodium acetate buffer pH 6.0, 100 μL of α-amyloglucosidase (3000 U/mL)) were added into samples, followed by a constant incubation at 37 °C for 300 min. Aliquots (0.1 mL) at 0, 5, 10, 20, 30, 45, 60, 90, 120, 180, 240, and 300 min were collected in 0.9 mL of 95% ethanol and the glucose content in aliquots was measured using a Megazyme GOPOD kit. Residues at 0, 20, and 120 min were sampled to analyze the phenolic contents. Each digestion test was repeated at least three times.

Rapidly digestible starch (*RDS*), slowly digestible starch (*SDS*), and resistant starch (*RS*) [32] were calculated as following Equations (4)–(6):

(4) $$\mathrm{RDS}\;(\%)=\frac{(G_{20}-F)\times 0.9}{T}\times 100$$

(5) $$\mathrm{SDS}\;(\%)=\frac{(G_{120}-G_{20})\times 0.9}{T}\times 100$$

(6) RS (%)=1−RDS (%)−SDS (%)

where G_20_ and G_120_ represent the glucose contents of hydrolysis within 20 and 120 min, respectively; F refers to the content of free glucose; T is the total weight of starch.

The hydrolysis kinetics of extruded noodles with unbound and bound phenolics, bound phenolics only were fitted to the first-order kinetic model [33] as shown in Equation (7):

(7) $$C_{t}=C_{\infty }\times (1-e^{-kt})$$

where C_t_ (%) is the percentage of starch in the noodles digested at time t (min), and C_∞_ (%) is the predicted digestibility at the end of the reaction; *k* (min^−1^) represents the rate coefficient of the enzyme hydrolysis. A logarithm of slope (LOS) plot (Equation (8)) was obtained by expressing the first derivative of the first-order equation in logarithmic form Equation (7).

(8) $$\ln\;(\frac{\mathrm{dc}}{\mathrm{dt}})=-kt+\ln(C_{\infty }k)$$

where $\ln\frac{\mathrm{dc}}{\mathrm{dt}}$ represents the logarithm of the slope, and the equation describes a linear relationship between LOS and time of hydrolysis, t (min). *k* and C_∞_ were used to construct model-fit starch digestion curves according to Equation (7).

### 2.6. Morphology

The morphologies of uncooked, cooked, and digested (0, 20, 120 min) noodles were characterized following the method of Wang, Li, Chen, Hui, Tang and Wei [13]. The noodles were lyophilized, fractured, and mounted on a copper stub. Afterward, samples were coated using a Sputter coater (JFC-1600, JEOL, Tokyo, Japan). The whole morphologies of the fracture surface of noodles were observed using a scanning electron microscope (SEM) (SU8000, HITACHI, Ltd., Tokyo, Japan) at a magnification of 30×.

### 2.7. Content of Unbound, Bound, Total Phenolics

Uncooked, cooked noodles and digestion residues (0, 20, and 120 min) were extracted three times with 80% methanol (1:5, *w*/*v*), followed by centrifugation at 2000× *g* for 10 min. The supernatants were collected and concentrated for the analysis of the unbound phenolic content (UPC), while the remaining precipitates were used to extract the bound phenolics. The precipitates were hydrolyzed in 4 mol/L sodium hydroxide solution (1:25, *w*/*v*) for 2 h (stirring, nitrogen circumstance). Then, the hydrolysates were centrifugated at 15,000× *g* for 30 min. The supernatants were concentrated for the analysis of bound phenolic content (BPC). The phenolic content was determined according to the description of Singleton and Rossi [34]. The intensity of blue was recorded at 760 nm after the reaction (2 h, room temperature) of blends of phenolic extracts, 0.2 N Folin-Ciocalteu reagent, and 7.5% sodium carbonate (1:5:4). Gallic acid was used as the standard. The results were expressed as ‘milligrams of gallic acid equivalents per gram of dry weight (mg GAE·g DW^−1^)’. Total phenolic content (TPC) was calculated by unbound phenolic content plus bound phenolic content.

### 2.8. Profiles of Unbound, Bound, Total Phenolics

The rutin, vitexin, and hyperin are predominant profiles in buckwheat hull extracts [25,35]. The rutin, vitexin, and hyperin standards were used to quantify their changing during in vitro digestion. A high-performance liquid chromatography (HPLC) analysis was performed using an Agilent 1260 (II) system (Agilent Technologies, Inc., Santa Clara, United States). A reverse phase C18 analytical column (4.6 mm × 250 mm, 5 μm, SuperLu C18 (2), Guangzhou FLM Scientific Instrument Co., Ltd, Guangzhou, China) was used for the separation of the profiles. The mobile phase consisted of A (0.1% phosphoric acid in water) and B (Acetonitrile). Gradient elution was programmed as follows: 0–9 min, 20% B; 9–14 min, 20–30% B; 14–19 min, 30–40% B; 19– 24 min, 40% B; 24–29 min, 40–20% B; followed by 5 min equilibration of 20% B. Phenolics samples (10 μL) were injected by an autosampler at a flow rate of 0.8 mL/min. The column temperature was set at 30 °C. The spectrogram was measured at 350 nm.

### 2.9. Bioaccessibility Index

The bioaccessibility index (*BI*) of the rutin, vitexin, and hyperin from phenolic-enhanced noodles represent the number of phenolic profiles released after simulated gastrointestinal digestion that could become available for absorption into the systemic circulation [36]. This index was determined as follows:

(9) $$\mathrm{BI}\;(\%)=\frac{C_{r}}{C_{0}}\times 100\;$$

where *C*_r_ is the total phenol content (mg/100 g) in digestion liquids after in vitro digestion, and *C*_0_ is the total phenol content (mg/100 g) before in vitro digestion.

### 2.10. Antioxidant Activity

Ferric reducing antioxidant power (FRAP) analysis was measured based on the method (Drinkwater, Tsao, Liu, Defelice, & Wolyn, 2015) to characterize the antioxidant activity of extruded noodles and residues during in vitro gastrointestinal digestion. FRAP reagent was prepared with a mixture of 300 μM Acetate buffer pH 3.6, 10 mM 2, 4, 6-Tris (2-pyridyl)-s-triazine (TPTZ) in 40 Mm HCl and 20 mM Ferric chloride (FeCl_3_) at a ratio of 10:1:1 (*v*/*v*/*v*). Then, 0.25 mL of the sample extract was mixed well with 2.5 mL of FRAP reagent. Then, the mixture was in incubation at 37 °C for 2 h, followed by the monitoring of absorbance at 593 nm using a spectrophotometer (V-1200, VWR, Leicestershire, UK). The iron (II) sulfate (FeSO_4_) (0 to 1000 μM) was prepared as the standard solution and assayed under similar conditions. FRAP results were expressed as ‘μmol of Fe^2+^ per gram dry weight (μmol Fe^2+^/g DW)’.

### 2.11. Statistical Analysis

All analysis was conducted in triplicate unless stated otherwise. ANOVA and Duncan’s multiple range test were used at a significance level of *p* < 0.05, accomplished using SPSS 18.0 (IBM, New York, NY, USA).

## 3. Results and Discussion

### 3.1. Content and Profiles of Phenolics of Raw Starch and Phenolic Extract

The extracted starch contained 0.463 mg GAE·g DW^−1^ of UPC, 0.160 mg GAE·g DW^−1^ of BPC, and 0.623 mg GAE·g DW^−1^ of TPC. The phenolic extract had a UPC of 284 mg GAE·g DW^−1^ without bound phenolics (Table S1). The three most predominant phenolic profiles in the buckwheat phenolic extract from the hull were rutin, vitexin, and hyperin [11,25], which were identified (Table S2) and quantified (Table S1). The starch extract had rutin (0.11 mg/100 g) only. The phenolic extract consisted of rutin (1674 mg/100 g), vitexin (528 mg/100 g), and hyperin (1114 mg/100 g). The phenolic extract had an excessively higher content of phenolics than the starch extract.

### 3.2. Characteristics of Starch-Phenolic Complexes Induced by Extrusion

**Figure 1:**
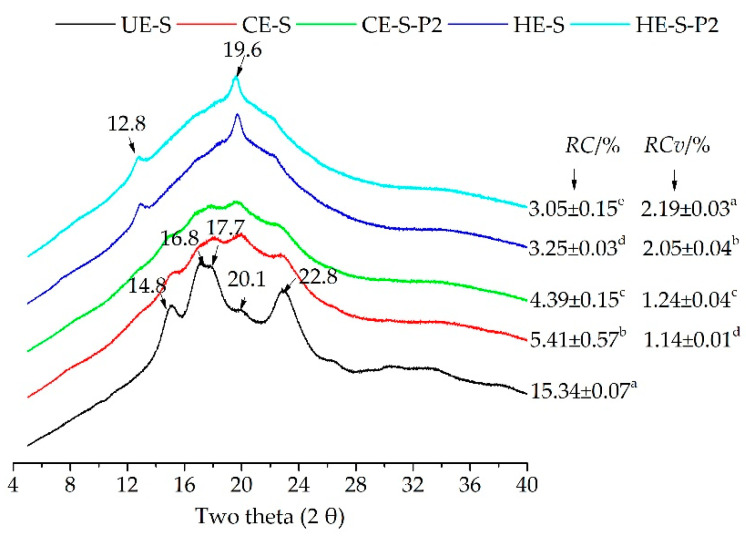
XRD pattern of native buckwheat starch, cold and hot extruded noodles with starch, and phenolic extracts. UE-S, unextruded (native) buckwheat starch; CE-S, cold extruded noodles with starch only; CE-S-P2, cold extruded noodles with starch and phenolics (2.0%); HE-S, hold extruded noodles with starch; HE-S-P2, hold extruded noodles with starch and phenolics (2.0%); *RC*, relative crystallinity; *RC*_v_, relative crystallinity of V-type crystallites. Different letters in a column represent values that are significantly different (*p* < 0.05).

XRD is an effective technique to characterize the formation of complexes between starch and phenolics, including non-inclusion complexes [37,38,39] or V-type inclusion complexes [40,41,42]. Compared with native starch with typical peaks of A-type crystallites (Figure 1), XRD patterns of cold extruded starch in the absence and presence of phenolics (CE-S and CE-S-P2) showed typical diffraction peaks of A+V-type crystallites with less pronounced peaks at 12.8°, 14.8°, 16.8°, 17.7°, 19.6° and 22.8° of 2θ (Figure 1) [43], indicating that the cold extrusion altered the starch crystalline structure, which is consistent with the results of the cold extruded wheat starch [44]. However, with the incorporation of phenolic compounds, the *RC* significantly decreased from 5.41% to 4.39% (*p* < 0.05). This suggests that phenolic compounds interfered with the crystalline structure of extruded starch. It is noted that the phenolic molecules caused a slight increase in the relative crystallinity of V-type crystallites (*RC*_v_), ranging from 1.14% to 1.24%. On the other hand, hot extrusion almost destroyed native A-type crystallites and induced the generation of new V-type crystallites. XRD patterns of hot extruded noodles with or without 2.0% of phenolics (HE-S and HE-S-P2) lost most peaks representing A-type crystallites, but sharp peaks at 12.8° and 19.6° of 2θ (V-type crystallites) appeared. Similar to CE noodles, the incorporation of phenolic compounds caused a decrease in *RC* from 3.25% to 3.05% and an increase in *RC*_v_ from 2.05% to 2.19%. Meanwhile, the proportion of V-type crystallites increased up to 72.1%. Hot extrusion caused lower *RC* but higher *RC*_v_ than cold extrusion. Tao, Zhu, Nan, Jiang and Wang [44] reported the consistent observation that the increased extrusion temperature caused a larger loss of relative crystallinity, which was associated with the thermal degradation of the amylopectin. Considering the disruption of the native crystalline structure and their recrystallization, especially the formation of V-type crystallites, the phenolic molecules might complex with amylopectin molecules in the crystalline region and amylose molecules in the amorphous region. Particularly, the semi-crystalline (type II) intrahelical V-type crystallites may result from the packed single helices of amylose and amylose-phenolic complexes [41]. Therefore, cold and hot extrusion may induce the formation of non-inclusion or inclusion complexes, and hot extrusion was likely to cause a higher intensity of complexation and more formation of V-type inclusion complexes between starch and phenolics compared to cold extrusion. This may interfere with the digestion of starch and the release of phenolics during digestion.

### 3.3. Simulated In Vitro Digestion of Extruded Noodles

Extruded phenolic-enhanced noodles showed a relatively low digestion rate and digestibility owing to the inhibitory effects of phenolics on the digestion enzymes (such as α-amylase) [11] or the possible interaction with starch, resistant to enzymatic hydrolysis [17]. The digestion properties of extruded noodles were compared to explore the roles of extrusion and phenolics in the digestion behavior of starch.

Compared with cold and hot extruded noodles, cooked unextruded starch showed a higher proportion of RDS (58.77%), a lower proportion of SDS (20.95%) and RS (20.28%), and a higher digestion rate (*k*) (0.0401 min^−1^) and digestibility (C_∞_) (98.34%). The main reason is attributed to the faster digestion of the powder samples (UE-S) than extruded noodle samples. Phenolic-enhanced noodles prepared through cold extrusion displayed a significantly lower proportion of RDS (24.12%) and SDS (39.60%) but a higher proportion of RS (36.28%) compared to noodles in the absence of phenolics (29.74%, 43.24%, and 27.02%, respectively) (*p* < 0.05) (Table 1). HE noodles showed a similar tendency to CE noodles in the presence or absence of phenolics (26.62%, 33.22%, and 40.15% vs. 34.75%, 37.08%, 28.17% for RDS, SDS, and RS, respectively). Of interest is that the former had a higher proportion of RDS and RS but lower SDS than the latter. The enzymatic hydrolysis curves (Figure S1) showed that the digestion was fast in the early stage and gradually slowed. Most of the starch (approximately 80%) was hydrolyzed after digestion for 120 min. The *k* values of phenolic-enriched noodles (0.0222 and 0.0175 min^−^^1^ for CE-S-P2 and HE-S-P2, respectively) decreased significantly when compared with the noodles with starch only (0.0272 and 0.0237 min^−1^ for CE-S and HE-S, respectively), indicating that phenolics caused the lower hydrolysis rate of starch after both cold and hot extrusion. Additionally, HE noodles had a lower hydrolysis rate of starch than CE noodles with corresponding formulas. With the incorporation of phenolics, decreased C_∞_ values were observed for CE and HE noodles (91.80–82.38% and 90.28–81.09%, respectively). In other words, the decreased digestibility was caused by phenolics.

Compared to cold extrusion, hot extrusion may cause a higher degree of gelatinization [14] and more degradation of starch [12,13], which possibly explains the increased RDS [45,46]. Chang, et al. [47] reported shorter chains with degrees of polymerization (DP) between 6 and 12 (A chains) of starch were digested more easily than the longer chains with DP of 24-48 (B_2_ and B_3_ chains). However, the leached amylose during extrusion may be responsible for the increase in RS [46]. The gelatinization process of starch is temperature dependent along with the amorphous swelling and disintegration of the crystal domain [48]. A hypothesis is that due to the limited disruption of the crystalline structure, a small number of phenolic molecules can complex with starch matrix in the amorphous region. As starch granules are completely gelatinized with a full disruption of crystalline structure, more phenolics will penetrate amorphous and crystalline regions, improving the complexation with amylose or amylopectin in the disrupted amorphous and crystalline regions. Jiang, et al. [49] reported a small amount of chlorogenic acid (approximately 1.5 mg/g of increase) was combined with the starch with a low degree of gelatinization at 65–70 °C. In contrast, more chlorogenic acid (approximately 3.5 mg/g of increase) complexed with fully gelatinized starch at 80–85 °C. With the incorporation of phenolics, HE starch with less crystalline structure (Figure 1) showed a higher possibility to complex with phenolics compared with CE starch, especially for the formation of V-type inclusion complexes with amylose, which are less susceptible to digestive, thus explained by a slow digestion rate and a low digestibility [41,50,51]. Therefore, the release of phenolics wrapped in the extruded starch matrix or interacting with extruded starch may have faced interference. It is noted that the soaking prior to cooking may affect the inner structure of CE and HE phenolic-enhanced noodles differently. The different degrees of crystallinity and V-type crystallites (Figure 1) may affect the water absorption in different manners, which in turn affects the starch structure [52] and the interaction of starch and guests [53]. The effects of soaking on the digestion of CE and HE extruded noodles are yet to be studied.

### 3.4. Morphology of Extruded Noodles and Residues during Digestion

**Figure 2:**
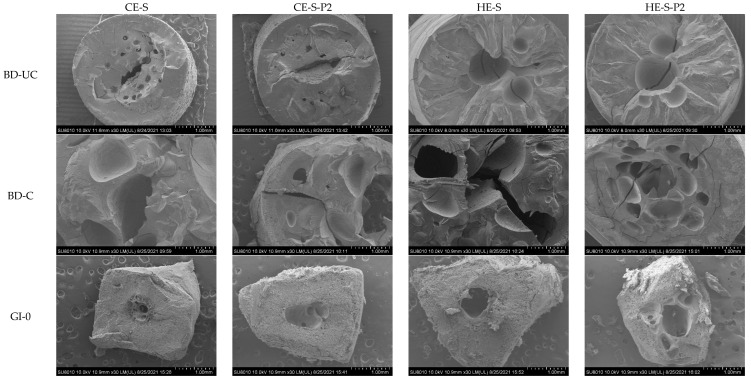

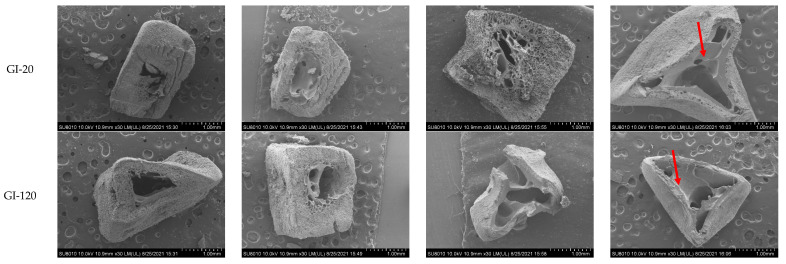
SEM images (30×) of the fracture surface of the cold and hot extruded noodles without or with phenolics before digestion (uncooked and cooked) and residues during simulated in vitro gastrointestinal digestion for 0, 20, and 120 min. CE-S, cold extruded noodles with starch only; CE-S-P2, cold extruded noodles with starch and phenolics (2.0%); HE-S, hold extruded noodles with starch only; HE-S-P2, hold extruded noodles with starch and phenolics (2.0%); BD-UC, uncooked noodles before digestion; BD-C, cooked noodles before digestion; GI-0, gastrointestinal digestion for 0 min (after gastric digestion); GI-20, gastrointestinal digestion for 20 min; GI-120, gastrointestinal digestion for 120 min.

Figure 2 illustrates the morphology of the cold and hot extruded noodles and residues during gastrointestinal digestion to visually observe the morphological changes in cooked and digested noodles. Compared to uncooked noodles, all cooked noodles displayed more irregular holes with the rough and discontinuous network, particularly in the inner noodles, which is consistent with our previous report [54], attributed to the penetration of water during cooking of noodles. After gastrointestinal digestion, intensive pores appeared in the outer noodles, and irregular holes became larger, particularly in the hot extruded samples, possibly attributed to acid hydrolysis of starch [47,55,56] or the physical or chemical breakdown under gastric acidic and shaking conditions [57]. Enzymatic hydrolysis of starch by α-amylase and α-amyloglucosidase into glucose units mainly occurs in the small intestine. The noodles gradually lost their original shape when starch was digested in the small intestine phase from 0 min to 120 min. Of note is that hot extruded phenolic-enhanced noodles seemed to have more matrix left (red arrows) after digestion, which was consistent with the lower digestibility of starch mentioned above.

### 3.5. Phenolic Contents of Extruded Noodles and Residues during Digestion

**Figure 3:**
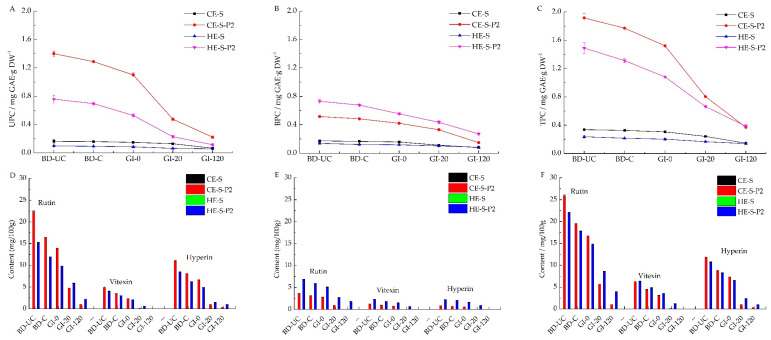
Contents of unbound (**A**), bound (**B**), and total (**C**) phenolics, and profiles in unbound (**D**), bound (**E**), and total (**F**) phenolic fractions of the cold and hot extruded noodles before digestion (uncooked and cooked) and residues during simulated in vitro gastrointestinal digestion for 0, 20, and 120 min. CE-S, cold extruded noodles with starch only; CE-S-P2, cold extruded noodles with starch and phenolics (2.0%); HE-S, hold extruded noodles with starch only; HE-S-P2, hold extruded noodles with starch and phenolics (2.0%); BD-UC, uncooked noodles before digestion; BD-C, cooked noodles before digestion; GI-0, gastrointestinal digestion for 0 min (after gastric digestion); GI-20, gastrointestinal digestion for 20 min; GI-120, gastrointestinal digestion for 120 min; UPC, BPC, and TPC, unbound, bound and total phenolic content respectively.

Before digestion, CE-S-P2 and HE-S-P2 contained 1.401 and 0.759 mg GAE·g DW^−1^ of UPC, respectively, significantly higher than uncooked CE-S (0.163 mg GAE·g DW^−1^) and HE-S (0.099 mg GAE·g DW^−1^) (Figure 3A). The cooking of noodles resulted in the loss of phenolic contents [58,59]. For instance, a decrease in UPC by 8.07% for CE-S-P2 and 8.30% for HE-S-P2 were observed. Cooking also significantly decreased the BPC of uncooked phenolic-enhanced noodles (0.515–0.483 and 0.731–0.678 mg GAE·g DW^−1^ for CE-S-P2 and HE-S-P2, respectively) (Figure 3B). The total phenolics content (TPC) was calculated through the sum of UPC and BPC (Figure 3C). Cooked CE-S-P2 and HE-S-P2 had a loss of 7.6% and 7.8% of TPC compared to the uncooked ones.

Along with gradual starch digestion, the phenolics on the surface, physically embedded and bound in the starch matrix after extrusion, can be released gradually. In turn, the released phenolics can inhibit starch digestion by interacting with digestion enzymes to inhibit enzyme activity, further interfering with the release of phenolics [7]. The release of phenolics from other components (such as protein) in starch extracts or phenolic degradation after release in gastric and small intestine conditions on phenolic content also affects degradation [11,60,61]. For instance, the phenolic content in digestion liquids was not detected. Instead, the release of unbound, bound, and total phenolic was monitored by measuring contents in residues of the noodles during digestion (Figure 3A–C).

There was a sustained drop in the UPC of noodles to a different extent at various stages of starch digestion (Figure 3A). The released UPC during gastric digestion was expressed as the difference in UPC between cooked noodles before digestion (BD-C) and residues after gastrointestinal digestion for 0 min (GI-0); the corresponding difference in UPC of residues between GI-0 and GI-20 or GI-20 and GI-120 represents the released UPC within the first 20 min or the following 100 min of small intestinal digestion. For phenolic-enhanced noodles, following the gastric digestion, unbound phenolics of CE-S-P2 and HE-S-P2 were released at 0.188 (14.6% of UPC in the cooked samples) and 0.167 (24.0%) mg GAE·g DW^−1^ respectively. At the early stage of small intestinal digestion (within 20 min), 0.625 (48.5%) and 0.301 (43.2%) mg GAE·g DW^−1^ of UPC were released from CE-S-P2 and HE-S-P2 residues, respectively; with gastrointestinal digestion from 20 min to 120 min (within 100 min), CE-S-P2 and HE-S-P2 residues released 0.254 (19.7%) and 0.113 (16.2%) mg GAE·g DW^−1^ of UPC respectively. Any remaining unbound phenolics (17.2% in CE-S-P2 and 16.6% in HE-S-P2) may be released completely as gastrointestinal digestion is prolonged. CE-S and HE-S noodles released 0.093 and 0.037 mg GAE·g DW^−1^ of UPC during the whole gastrointestinal digestion. The phenolic-enhanced noodles released the highest proportion of unbound phenolics within the first 20 min of small intestinal digestion, consistent with the result of Chi, Li, Zhang, Chen, Xie, Li and Bai [5], who reported the drastically increased release of gallic acid in the initial stage. Hot extruded noodles enhanced with phenolics released more unbound phenolics in the gastric phase than cold extruded noodles with corresponding formulas within the same stage of digestion. Meanwhile, the cold extruded noodles released more in the small intestinal phase than cold extruded samples, which was consistent with the corresponding starch digestion (higher proportion of digested starch for cold extruded noodles before 120 min gastrointestinal digestion).

The bound phenolics content (BPC) experienced a gradual release during starch digestion (Figure 3B), which was different from the unbound fractions. In the gastric phase, CE-S-P2 and HE-S-P2 released 12.8% and 18.1% of BPC, respectively; following the first 20 min of small intestinal digestion, 19.0% and 17.6% of BPC were released from the CE-S-P2 and HE-S-P2 residues respectively. Afterward, within the later 100 min of small intestinal digestion, the CE-S-P2 and HE-S-P2 residues were released at 37.5% and 24.3% of BPC, respectively. Bound phenolics remaining in noodle residues (30.7% in CE-S-P2 and 40% in HE-S-P2) may be further released as digestion undergoes or are carried into the large intestine to release. CE-S and HE-S noodles released 0.089 and 0.040 mg GAE·g DW^−1^ of BPC during the whole gastrointestinal digestion. Different from the release of unbound phenolics, both the cold and hot extruded phenolic-enhanced noodles released the highest proportion of bound phenolics within the later 100 min of small intestinal digestion during whole gastrointestinal digestion. Hot extruded phenolic-enhanced noodles displayed a more controlled release of bound phenolics in the gastric and small intestinal phase than cold extrusion. Hot extruded phenolic-enhanced noodles released more bound phenolics in the gastric phase, whereas cold extruded noodles released more in the small intestinal phase. After 120 min of gastrointestinal digestion, a larger proportion of bound phenolics (slower release) remained in residues than unbound phenolics may be attributed to the protection of starch-phenolic complexes [6].

In terms of the changes in total phenolics content (TPC) during digestion (Figure 3C), CE-S-P2 and HE-S-P2 after gastrointestinal digestion for 120 min released 79.2% and 71.9%, respectively, consisting of 14.1% and 21.2% in gastric digestion, 40.5% and 30.6% in the first 20 min of gastrointestinal digestion, and 24.6% and 20.1% in the late 100 min of gastrointestinal digestion, respectively. This indicated that the extrusion treatment could be used to control the release of phenolics in starchy foods enriched in phenolics and that hot extrusion may be better than cold extrusion. 

### 3.6. Phenolic Profiles of Extruded Noodles and Residues during Digestion

The changes of rutin, vitexin, and hyperin in the unbound, bound, and total phenolic fractions of extruded noodles and their residues during in vitro gastrointestinal were monitored (Figure 3D–F). Among the three phenolics, rutin was the most abundant profile in unbound, bound, and total phenolic fractions. In addition, phenolic profiles in all phenolic fractions of CE-S and HE-S before and after digestion were not detected. Therefore, the variation of rutin content in phenolic-enhanced noodles (CE-SP2 and HE-S-P2) during whole digestion was mainly analyzed.

Before digestion, in the unbound phenolic fraction (Figure 3D), the rutin contents of uncooked CE-S-P2 and HE-S-P2 were 22.42 and 15.23 mg/100 g samples, respectively. Rutin contents of cooked CE-S-P2 and HE-S-P2 decreased to 16.41 and 11.92 mg/100 g, respectively, with a loss of 26.8% for CE-S-P2 and 21.7% for HE-S-P2. Cooking also resulted in a significant decrease of rutin content in the bound phenolic fraction from 3.58 and 6.85 mg/100 g for the uncooked CE-S-P2 and HE-S-P2 to 3.11 and 5.88 mg/100 g for the cooked ones, with a loss of 13.1% and 14.2%, respectively (Figure 3E). Total rutin content (the sum of rutin content in unbound and bound phenolic fractions) of uncooked CE-S-P2 and HE-S-P2 decreased by 24.5% and 19.6% after cooking, respectively (Figure 3F).

In general, in the unbound fraction, rutin contents experienced a significant decrease for both CE-S-P2% and HE-S-P2% during gastrointestinal digestion, indicating the increased release of rutin (Figure 3D). Following the gastric digestion, rutin of CE-S-P2 or HE-S-P2 was released from 16.41 to 13.95 mg/100 g (15.0%) or from 11.92 to 9.77 mg/100 g (18.0%), respectively. During small intestinal digestion for 20 min, 56.1% or 32.6% of rutin were released from the CE-S-P2 and HE-S-P2 residues, respectively. As digestion went from 20 min to 120 min (within 100 min), CE-S-P2 and HE-S-P2 residues released 22.9% and 31.0% of rutin, respectively. The remaining rutin in the unbound fraction (6.0% for CE-S-P2 and 18.4% for HE-S-P2) may be released completely with prolonged gastrointestinal digestion. Hot extruded phenolic-enhanced noodles displayed a slower release of rutin in the unbound fraction during digestion than cold extrusion, which may be explained by better embedment in the starch matrix [7].

The release of rutin in the bound fraction behaved differently from that in the unbound fractions (Figure 3E). In the gastric phase, CE-S-P2 and HE-S-P2 released 10.3% and 14.3% of rutin, respectively. Following the first 20 min of small intestinal digestion, 60.1% and 40.3% of rutin were released from the CE-S-P2 and HE-S-P2 residues, respectively. Afterward, CE-S-P2 and HE-S-P2 residues within the later 100 min of small intestinal digestion released 29.6% and 15.5% of rutin, respectively. CE-S-P2 released rutin almost completely, whereas HE-S-P2 digestion residues remained at 29.9% of bound rutin, some of which might be further released with extended digestion or carried into the large intestine (V-type inclusion complexes considered as resistant starch [62]). Zhu, Liu, Wang, Huang, Zhong and Li [7] reported that epigallocatechin-3-O-gallate (EGCG) embedded on the surface of starch was released rapidly in the first h, EGCG embedded in the cross-section of starch granule was released slowly in 1–3 h, and the EGCG complexed with amylose and amylopectin were finally released and reached equilibrium after 3 h. Therefore, the slower release of rutin in the bound fraction of HE-S-P2 than CE-S-P2 might be owing to the higher complexation intensity of rutin with amylose or amylopectin during gelatinization induced by hot extrusion [37]. The higher release of rutin within 20 min of small intestinal digestion than in other digestion stages may be because rutin was likely unable to form inclusion complexes with starch.

CE-S-P2 and HE-S-P2, after gastrointestinal digestion for 120 min, had a cumulative release of total rutin of 94.88% and 77.93%, respectively (Figure 3F). As a result, hot extrusion may be better to assist in controlling the release of rutin in the starch matrix than cold extrusion.

Apart from the rutin, the vitexin and hyperin in unbound, bound, and total phenolic fractions of CE-S-P2 and HE-S-P2 showed a similar sustained release to rutin during in vitro gastrointestinal digestion. Differently, vitexin and hyperin can be released completely earlier than rutin; for instance, these profiles in the bound fraction could not be detected after gastrointestinal digestion for 20 min to CE-S-P2 and 120 min to HE-S-P2. This may be attributed to the complexation between rutin, vitexin, or hyperin and extruded starch. Owing to different structures and conformation among three phenolic profiles (Figure S2), they may complex with starch with different intensities and manners. For instance, rutin possesses more hydroxyl groups than vitexin and hyperin, which may have more complexation with extruded starch [63]. Hydroxyl groups of phenolics have been reported to complex with starch through the hydrogen [64] or the CH-π [65] bonds. However, the experimental proof of the binding differences between rutin, vitexin, hyperin, and extrude starch is limited.

### 3.7. Bioaccessibility Index of Main Phenolic Profiles

The bioaccessibility of three major phenolic profiles (rutin, vitexin, and hyperin) from phenolic-enhanced noodles was evaluated based on the bioaccessible content of profiles in the digestion liquids after in vitro gastrointestinal digestion (Table 2). The bioaccessibility index of rutin in digestion liquids (66.50% for CE-S-P2 and 65.81% for HE-S-P2) was higher than those of vitexin and hyperin. However, the total release of rutin of CE-S-P2 after gastrointestinal digestion based on residues (Figure 2F) was higher than HE-S-P2. Still, similar bioaccessibility was observed, which was attributed to more degradation loss of rutin (5.55 mg/g) after faster release from CE-S-P2 than that from HE-S-P2 (2.16 mg/g). Additionally, HE-S-P2 remained at 22.07% of total rutin, which was higher than CE-S-P2 (5.12%). The remaining rutin was released as prolonged digestion in the small intestine or fermentation in the colon, suggesting that hot extrusion may protect more potential bioaccessible rutin. Different phenolic acids and flavonoids have been shown by an increased or decreased bioaccessibility index after in vitro gastrointestinal digestion, in which the rutin showed a bioaccessibility index of 267.2% [36]. The reason for this higher bioaccessibility value is due to their increased release from conjugated and bound forms after in vitro digestion [36]. In our studies, rutin showed a relatively low bioaccessibility, as the rutin in phenolic extracts was in the free form rather than the conjugated and bound forms.

### 3.8. Antioxidant Activity of Extruded Noodles and Residues during Digestion

Phenolic compounds are considered effective antioxidant ingredients for improving foods’ antioxidant activity, which is usually consistent with phenolic contents [59,66]. Compared to noodles in the absence of phenolics (CE-S and HE-S), phenolic-enhanced noodles (CE-S-P2 and HE-S-P2) at each stage showed significantly increased FRAP (*p* < 0.05). As phenolics were lost during cooking and released during digestion, phenolic contents of extruded noodles and residues gradually decreased, resulting in decreased FRAP (Table 3).

Concerning FRAP of unbound phenolics, uncooked CE-S-P2 and HE-S-P2 showed higher FRAP (19.088 and 9.581 μmol Fe^2+^·g DW^−1^, respectively) compared to the corresponding cooked samples (16.609 and 7.882 μmol Fe^2+^·g DW^−1^). As digested continuously, CE-S-P2 witnessed a significant decrease in FRAP (16.609-13.708-5.474-2.884 μmol Fe^2+^·g DW^−1^), and similar tendency but lower values were observed for HE-S-P2 (7.882-7.413-2.405-1.070 μmol Fe^2+^·g DW^−1^). On the other hand, the bound phenolic fractions of uncooked CE-S-P2 and HE-S-P2 were reported with 6.665 and 8.222 μmol Fe^2+^·g DW^−1^ of FRAP, respectively. This was significantly higher than that of cooked CE-S-P2 (5.775 μmol Fe^2+^·g DW^−1^) and HE-S-P2 (7.452 μmol Fe^2+^·g DW^−1^). The FRAP of bound phenolic fractions of digested CE-S-P2 and HE-S-P2 significantly decreased to 1.335 and 3.002 μmol Fe^2+^·g DW^−1^, respectively. Hot extruded phenolic-enhanced noodles consisted of a higher content of bound phenolics than cold extruded noodles (Figure 3B), which was responsible for a higher FRAP.

### 3.9. Possible Mechanism of Unbound and Bound Phenolic Release

**Figure 4:**
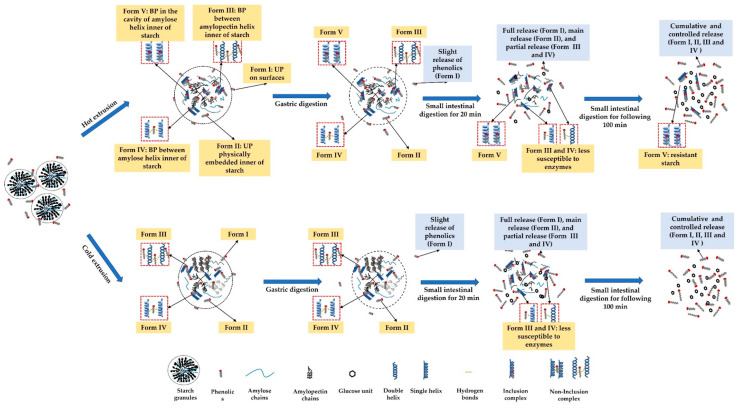
Possible releasing mechanism of unbound and bound phenolics from the extruded noodles during simulated in vitro gastrointestinal digestion.

The phenolics in extruded starchy noodles may exist in unbound and bound forms, that is, unbound phenolics embedded on the surfaces of the starch matrix (form I) and physically embedded inner starch matrix (Form II), as well as bound phenolics forming non-inclusion complexes with amylopectin (form III) or amylose (Form IV) or inclusion complexes with amylose (Form V) (Figure 4). The structural characteristics need to be identified.

Extrusion can disrupt the multi-level structure of starch (i.e., crystalline structure and chain distributions) [67], with starch gelatinization, melting, and fragmentation [68] owing to thermal and mechanical effects [12]. In general, the higher the degree of gelatinization of starch, the higher the complexation intensity between starch and phenolics. For instance, a higher intensity of complexation between quercetin and starch was observed when the blends were gelatinized at a temperature of 100 °C (higher degree of gelatinization) than that at 70 and 80 °C (lower degree of gelatinization) [32]. Therefore, compared to cold extrusion (40 °C), hot extrusion (90 °C) is more likely to induce more phenolics to complex with degraded or gelatinized starch during extrusion, resulting from a higher degree of gelatinization [14], particularly forming more inclusion complexes with leached amylose [41].

The unbound phenolics embedded on the surfaces of the starch matrix (form I) and physically embedded inner starch matrix (Form II) are preferentially released compared to bound phenolics (Form III, IV, and V) (Figure 4), as reported by Zhu, Liu, Wang, Huang, Zhong and Li [7]. This explained the varying release of UPC of extruded noodles compared to that of BPC, with a higher release during simulated gastrointestinal digestion for 120 min (cumulative release exceeding 80% vs. 60%) (Figure 3A). Of interest is that the hot extruded samples released higher UPC and BPC during gastro digestion but lower ones during small intestinal digestion. This may be because: (1) during gastro digestion, the structural differences of noodles or pasta can affect the rate of diffusion and surface erosion by digestive fluids [55,69]. HE noodles displayed less crystalline structure (Figure 1) and microstructure with larger irregular holes after cooking (Figure 2), which may absorb more gastric digestive fluids during the simulated gastric digestion phase (pH 1~2). The noodle structure and the starch within the noodles may be hydrolyzed more easily by the acidic solution compared with CE noodles and release higher amounts of phenolics; (2) during small intestinal digestion, because the digestion was mainly influenced by enzyme hydrolysis, hot extruded noodles contained more bound phenolics that are less susceptible to enzymes, on the other hand, more released phenolics during gastro digestion may inhibit the enzyme activity, thereby, explaining the lower digestibility of starch (Table 1) releasing fewer phenolics (Figure 3A,B). Owing to the V-type inclusion complex, this can sometimes be considered as resistant starch [62]; for instance, in higher resistant starch (HE-S-P2) (Table 1), phenolics included in the cavity of amylose helix (Form V) may be scarcely released during gastro-small intestinal digestion. This might explain why there are more remaining bound phenolics in hot extruded noodles than in cold extruded samples (Figure 3B). However, the relationship between the complexation between starch and phenolics and the release of phenolics during digestion needs further evidence.

## 4. Conclusions

More unbound phenolics (>80%) and less bound ones (~60%) were released after simulated in vitro gastrointestinal digestion, respectively. A rational design of starch-phenolic complexes can control the release of phenolics and further improve their bioavailability during gastrointestinal digestion. Compared with cold extrusion, hot extrusion induced the formation of more starch-phenolic complexes, especially the V-type inclusion complexes, with a higher proportion of bound phenolics, contributing to slower starch digestion and, thus, a more controlled release of phenolics. A lower proportion of BPC (41.9%) was released from hot extruded noodles than from cold extruded noodles (56.5%). Particularly, a lower proportion of complexed rutin was released from CE-S-P2 into the simulated small intestinal phase than that from HE-S-P2 (89.7% vs. 55.8%). Hot extrusion may allow more potential bioaccessible phenolics (such as rutin) than cold extrusion. Further studies must focus on the relationship between the dynamic structural changes of starch-phenolic complexes and phenolic release in vitro and in vivo gastrointestinal tracts.

## Figures and Tables

**Table 1:** Simulated in vitro gastrointestinal digestion properties of cold and hot extruded noodles with or without phenolics.

Samples	RDS (%)	SDS (%)	RS (%)	*k* (min^−1^)	*C_∞_* (%)
UE-S	58.77 ± 2.10 ^a^	20.95 ± 0.53 ^e^	20.28 ± 1.58 ^e^	0.0401 ± 0.0015 ^a^	98.34 ± 0.47 ^a^
CE-S	29.74 ± 0.78 ^c^	43.24 ± 1.71 ^a^	27.02 ± 0.94 ^d^	0.0272 ± 0.0011 ^b^	91.80 ± 1.54 ^b^
CE-S-P2	24.12 ± 0.88 ^e^	39.60 ± 0.49 ^b^	36.28 ± 1.37 ^b^	0.0222 ± 0.0005 ^d^	82.38 ± 1.43 ^c^
HE-S	34.75 ± 0.85 ^b^	37.08 ± 0.69 ^c^	28.17 ± 0.15 ^c^	0.0237 ± 0.0006 ^c^	90.28 ± 0.55 ^b^
HE-S-P2	26.62 ± 1.24 ^d^	33.22 ± 0.99 ^d^	40.15 ± 0.25 ^a^	0.0175 ± 0.0003 ^e^	81.09 ± 0.25 ^c^

UE-S, unextruded starch powder (native); CE -S, cold extruded noodles with starch only; CE-S-P2, cold extruded noodles with starch and phenolics (2.0%); HE-S, hold extruded noodles with starch only; HE-S-P2, hold extruded noodles with starch and phenolics (2.0%); RDS, rapidly digestive starch; SDS, slowly digestive starch; RS, resistant starch. Significant differences in digestion parameters of extruded noodles are expressed by different lowercase letters in the same column (*p* < 0.05).

**Table 2:** Bioaccessibility index of main phenolic profiles in digestion liquids of cold and hot extruded noodles during simulated in vitro gastrointestinal digestion.

Noodles	Profiles	*C*_0_ (mg/100 g)	*C*_g_ (mg/100 g)	*C*_i_ (mg/100 g)	*BI*_g_ (%)	*BI*_i_ (%)
CE-S	Rutin, vitexin, hyperin	n.d.	n.d.	n.d.	n.d.	n.d.
CE-S-P2	Rutin	19.55 ± 0.10 ^a^	1.77 ± 0.01 ^a^	13.00 ± 0.06 ^b^	9.05	66.50
	vitexin	4.39 ± 0.08 ^f^	0.71 ± 0.01 ^e^	1.42 ± 0.01 ^c^	16.17	32.35
	hyperin	8.83 ± 0.11 ^c^	1.21 ± 0.00 ^c^	2.87 ± 0.01 ^e^	13.70	32.50
HE-S	Rutin, vitexin, hyperin	n.d.	n.d.	n.d.	n.d.	n.d.
HE-S-P2	Rutin	17.81 ± 0.10 ^b^	1.39 ± 0.02 ^b^	11.72 ± 0.07 ^a^	7.80	65.81
	vitexin	4.79 ± 0.15 ^e^	0.52 ± 0.02 ^f^	1.32 ± 0.04 ^d^	10.86	27.56
	hyperin	8.21 ± 0.10 ^d^	1.07 ± 0.01 ^d^	2.86 ± 0.01 ^e^	13.03	34.84

*C*_0_, the content of phenolic profiles before digestion; *C*_g_, the content of phenolic profiles in gastric digestion liquid; *C*_i_, the content of phenolic profiles in small intestinal digestion liquid (120 min); *BI*_g_, bioaccessibility index of profiles in gastric digestion liquid; *BI*_i_, bioaccessibility index of profiles in small intestinal digestion liquid; n.d., not detected. Different letters in a column represent values that are significantly different (*p* < 0.05).

**Table 3:** Antioxidant activity (FRAP, μmol Fe^2+^·g DW^−1^) of the cold and hot extruded noodles with or without phenolics and residues during simulated in vitro gastrointestinal digestion (0, 20, and 120 min).

Noodles	Unbound Phenolics					Bound Phenolics				
	BD-UC	BD-C	GI-0	GI-20	GI-120	BD-UC	BD-C	GI-0	GI-20	GI-120
CE-S	1.588 ± 0.078 ^aC^	1.535 ± 0.238 ^abC^	1.312 ± 0.022 ^bC^	1.171 ± 0.046 ^cC^	0.356 ± 0.016 ^dC^	1.625 ± 0.107 ^aC^	1.571 ± 0.053 ^aC^	1.489 ± 0.033 ^aC^	0.922 ± 0.035 ^bC^	0.522 ± 0.023 ^cD^
CE-S-P2	19.088 ± 0.822 ^aA^	16.609 ± 0.850 ^bA^	13.708 ± 1.666 ^cA^	5.474 ± 0.098 ^dA^	2.884 ± 0.126 ^eA^	6.665 ± 0.209 ^aB^	5.775 ± 0.138 ^bB^	5.035 ± 0.224 ^cB^	3.686 ± 0.362 ^dB^	1.335 ± 0.021 ^eB^
HE-S	0.959 ± 0.036 ^aD^	0.912 ± 0.013 ^aD^	0.759 ± 0.032 ^bD^	0.416 ± 0.030 ^cD^	0.225 ± 0.030 ^dD^	1.235 ± 0.210 ^aD^	1.166 ± 0.065 ^aD^	1.002 ± 0.032 ^bD^	0.909 ± 0.012 ^cC^	0.669 ± 0.044 ^dC^
HE-S-P2	9.581 ± 0.037 ^aB^	7.882 ± 0.543 ^bB^	7.413 ± 0.779 ^cB^	2.405 ± 0.254 ^dB^	1.070 ± 0.046 ^eB^	8.222 ± 0.613 ^aA^	7.452 ± 0.448 ^bA^	6.515 ± 0.201 ^cA^	4.673 ± 0.387 ^dA^	3.002 ± 0.552 ^eA^

CE-S, cold extruded noodles with starch only; CE-S-P2, cold extruded noodles with starch and phenolics (2.0%); HE-S, hold extruded noodles with starch; HE-S-P2, hold extruded noodles with starch and phenolics (2.0%); BD-UC, uncooked noodles before digestion; BD-C, cooked noodles before digestion; GI-0, gastrointestinal digestion for 0 min (after gastric digestion); GI-20, gastrointestinal digestion for 20 min; GI-120, gastrointestinal digestion for 120 min. Significant differences in FRAP of unbound or bound phenolic fractions during gastrointestinal digestion are expressed by different lowercase letters in the same row (*p* < 0.05); Significant differences in FRAP among extruded noodles are expressed by different capital letters in the same column (*p* < 0.05).

## Data Availability

Research data are not publicly available.

## Supplementary Materials

The following supporting information can be downloaded at: https://www.mdpi.com/article/10.3390/nu14183864/s1, Figure S1: In vitro digestion curve of CE (A) and HE (B) starch noodles in the absence or presence of phenolics; Figure S2: Structure of rutin (A), vitexin (B), and hyperin (C) downloaded from PubChem (https://pubchem.ncbi.nlm.nih.gov/ (accessed on 5 August 2022)); Table S1: Phenolic content and main profiles of native starch and phenolic extracts; Table S2: Predominant phenolic profiles in buckwheat hull extract determined by a liquid chromatography electrospray ionization mass spectrometry (LC-ESI-MS).
