# Supplementary material for: Phenolic Release during In Vitro Digestion of Cold and Hot Extruded Noodles Supplemented with Starch and Phenolic Extracts
Source: Nutrients. 2022 Sep 18;14(18):3864. doi: 10.3390/nu14183864 (PMC9504551; doi:10.3390/nu14183864)
Supplement: Supplementary file 1 [file nutrients-14-03864-s001.zip › nutrients-1878911 - Supplementary Materials version 2.pdf]

nutrients

MDPI

Supplementary Materials

Phenolic Release during In Vitro Digestion of Cold and Hot Extruded Noodles Supplemented with Starch and Phenolic Extracts

Ruibin Wang [1,2,3], Ming Li [1,†], Margaret Anne Brennan [2], Don Kulasiri [2], Boli Guo [1,*] and Charles Stephen Brennan [3,4,*]

[1] Key Laboratory of Agro-Products Processing, Institute of Food Science and Technology, Chinese Academy of Agriculture Sciences, Ministry of Agriculture and Rural Affairs, Beijing 100193, China
[2] Department of Wine, Food and Molecular Biosciences, Faculty of Agriculture & Life Sciences, Lincoln University, Lincoln 7647, New Zealand
[3] Riddet Institute, Massey University, Palmerston North 4474, New Zealand
[4] School of Science, Royal Melbourne Institute of Technology University, Melbourne, VIC 3000, Australia
* Correspondence: guoboli@caas.cn (B.G.); charles.brennan@rmit.edu.au (C.S.B.)
† These authors contributed equally to this work.

Supplementary information

Table S1. Phenolic content and main profiles of native starch and phenolic extracts.

Materials	Phenolic content (mg GAE·g DW^{-1})			Main phenolic profiles in unbound phenolic extract (mg/100g)		
	UPC	BPC	TPC	Rutin	Vitexin	Hyperin
Starch	0.463 ± 0.039	0.160 ± 0.013	0.623 ± 0.045	0.139 ± 0.001	n.d.	n.d.
Phenolics	284 ± 13	n.d.	284 ± 13	1674 ± 24	528 ± 74	1114 ± 28

UPC, BPC and TPC, unbound, bound and total phenolic content, respectively; n.d., not detected.

Table S2. Predominant phenolic profiles in buckwheat hull extract determined by a liquid chromatography electrospray ionization mass spectrometry (LC-ESI-MS).

Profile	Molecular formula	Selected ion	m/z	MS2
Vitexin/isovitexin	$C_{21}H_{19}O_{10}$	$[M-H]^-$	431.09848	311.05634; 283.06128;
Hyperin	$C_{21}H_{19}O_{12}$	$[M-H]^-$	463.08884	300.02768; 271.02496; 151.00244; 178.99753
Rutin	$C_{27}H_{29}O_{16}$	$[M-H]^-$	609.12692	300.03;151.00;179.00;

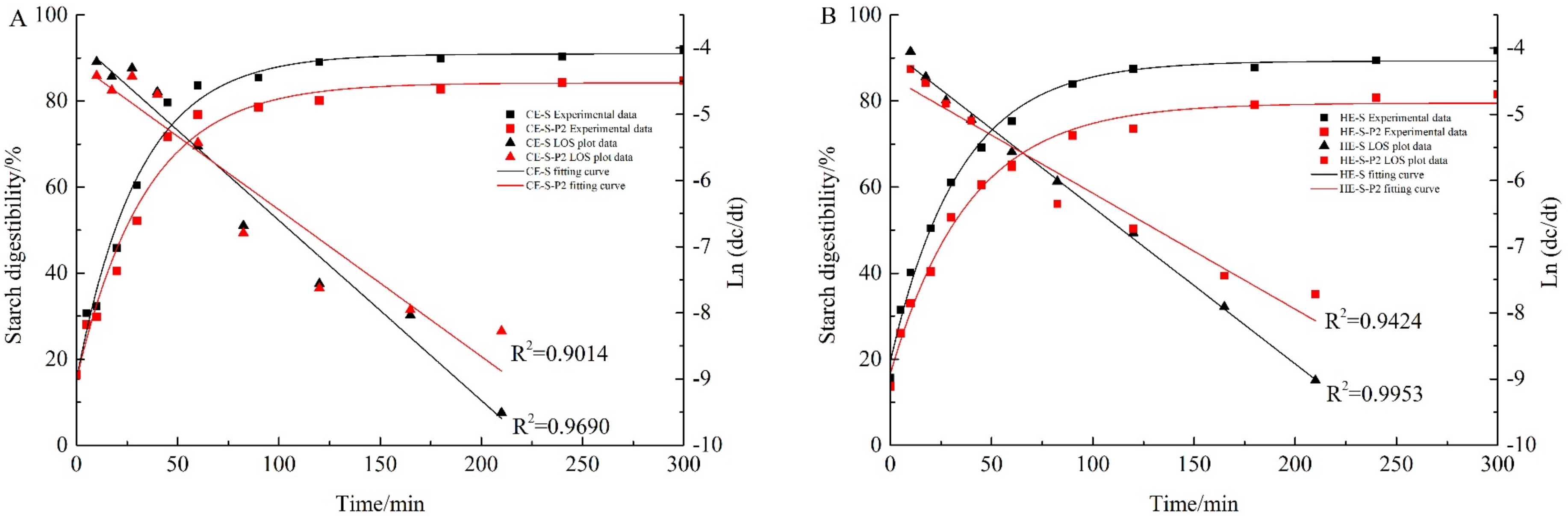

Figure S1. *In vitro* digestion curve of CE (A) and HE (B) starch noodles in the absence or presence of phenolics. CE-S, cold extruded noodles with starch only; CE-S-P2, cold extruded noodles with starch and phenolics (2.0%); HE-S, hold extruded noodles with starch only; HE-S-P2, hold extruded noodles with starch and phenolics (2.0%).

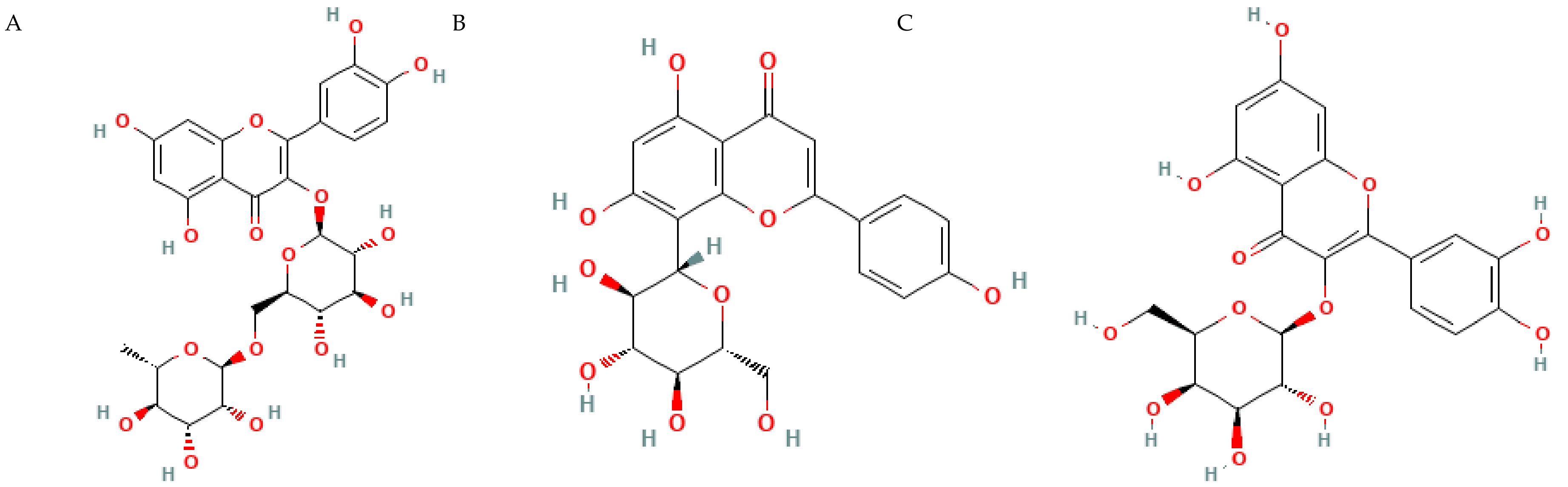

Figure S2. Structure of rutin (A), vitexin (B), and hyperin (C) downloaded from PubChem (https://pubchem.ncbi.nlm.nih.gov/).
